# 亚肺叶切除术治疗早期非小细胞肺癌时肺切缘的研究进展

**DOI:** 10.3779/j.issn.1009-3419.2018.06.11

**Published:** 2018-06-20

**Authors:** 宗凯 王, 明建 葛

**Affiliations:** 400016 重庆，重庆医科大学附属第一医院胸外科 Department of Thoracic Surgery, The First Affiliated Hospital of Chongqing Medical University, Chongqing 400016, China

**Keywords:** 亚肺叶切除术, 肺肿瘤, 早期, 肺切缘, 局部复发, Sublobar resection, Lung neoplasms, Early-staged, Surgical margin, Local recurrences

## Abstract

近年来随着低剂量计算机断层扫描(low-dose computed tomography, LDCT)、高分辨率CT(high-resolution CT, HRCT)的普及, 早期非小细胞肺癌的发现比率不断上升, 越来越多的胸外科医生探索手术方式的改良, 推动手术切除范围向更加合理的方向进展。临床研究发现肺切缘阴性较阳性有更低的局部肿瘤复发率, 因此亚肺叶切除术治疗早期肺癌术中保证肺切缘阴性具有重要的临床意义, 本文将就这一领域研究现状和进展作一综述。

## Abstract (en)

In recent years, with the popularization of low-dose computed tomography (LDCT) and high-resolution CT (HRCT), the discovery rate of early-staged non-small cell lung cancer has been on the rise, and more thoracic surgeons have explored more reasonable resection scope.Clinical studies have demonstrated that there is a lower rate of local tumor recurrence in patients with negative lung margins compared with positive ones.Therefore, it is of great clinical significance to ensure the negative margin during sublobar resection for early-staged lung cancer.This paper will focus on this area.

国家癌症中心发布的2017中国最新癌症数据显示, 肺癌已成为我国男女癌症患者死亡的首要原因^[1]^。随着对高危人群使用低剂量计算机断层扫描(low-dose computed tomography, LDCT)筛查和高分辨率CT(high-resolution CT, HRCT)的应用, 早期非小细胞肺癌(non-small cell lung cancer, NSCLC)的发现比率与日俱增, 越来越多的胸外科医生选择手术风险更低的亚肺叶切除(肺段或肺楔形切除术), 而肺切缘的质量与肿瘤的复发间的关系则少有研究。胸外科医生在选择亚肺叶切除术治疗早期肺癌过程中, 肺切缘的质量需要引起重视。关于切缘距离是否影响预后, 目前仍然没有定论^[2]^。在行亚肺叶切除术时, 切缘距离由外科医生在术中决定, 而切缘距离正是决定切缘质量的重要因素。如何在保留更多健康肺组织的前提下保证切缘阴性, 仍待进一步的研究, 本文将就此领域的研究进展进行综述。

## 1 早期NSCLC是否可以选择亚肺叶切除

早期NSCLC治疗的金标准为肺叶切除术, 同时行纵隔淋巴结清扫或系统性淋巴结采样^[3]^。这项标准背后的大部分证据是基于肺癌研究小组(Lung Cancer Study Group, LCSG)完成的一项随机对照试验, 试验结果显示与肺叶切除组相比, 亚肺叶切除组局部复发率增加3倍, 术后总体生存率(overall survival, OS)略有下降^[4]^。而这个试验却没有对局部复发是否与切缘阳性相关进行深究。早在1980年Hoffmann等^[5]^提出Ⅰ期无转移迹象的周围型NSCLC进行楔形切除术与肺叶切除术相比也可获得良好生存期。然而, 1995年Ginsberg等^[4]^公布了针对T1N0M0 NSCLC的随机对照试验结果, 结果显示亚肺叶切除与肺叶切除相比局部复发率有所提高。因此推荐将肺叶切除术作为早期肺癌的标准术式。随着检查手段的进步, 越来越多的Ia期NSCLC被早期发现^[6-8]^。HRCT的广泛应用, 早期发现以磨玻璃影(ground glass opacity, GGO)为主要成分的肿瘤增多^[8]^。因此对患者行肺叶切除术还是亚肺叶切除术成为近年来业内讨论热点。在一项包含16, 819例患者的临床大样本研究中发现, 对于1.0 cm或更小肿瘤患者, 行肺叶、肺段或楔形切除术疾病特异性生存率无统计学差异; 对于1.1 cm-2.0 cm的肿瘤, 肺叶切除术和肺段切除术具有相同的生存率, 比楔形切除具有更高生存率; 对于2.1 cm-3.0 cm的肿瘤, 肺叶切除仍然是标准的手术程序; 对于肺叶切除不适合的患者, 肺段切除和楔形切除显示相似的生存率^[9]^。美国国立综合癌症网络(National Comprehensive Cancer Network, NCCN)指南也推荐将肺段切除术意向性地运用于以下肺癌患者之一:原位腺癌; GGO成分大于50%;肿瘤倍增时间大于400 d。

多项回顾性研究显示了肺叶切除术与亚肺叶切除术之间的生存率和复发率差异, 并证明存在相互矛盾的结果^[10-13]^。澳大利亚悉尼麦格理大学协作研究(CORE)小组的曹等^[14]^进行了一项系统评价和荟萃分析, 试图调和以前研究的矛盾结果, 共有来自6个在线数据库的54项研究, 得出的主要结论是, 当选择适当的患者时, 亚肺叶切除术, 特别是肺段切除术, 具有相似的总体和无病存活率。为了确定那些潜在适合亚肺叶切除的患者, 比较后:肿瘤 < 2 cm; 边缘距离至少2 cm-3 cm; 高分辨率CT显示高比例的GGO可以作为亚肺叶切除术的标准。在回顾性研究中, 许多挑战阻碍了肺叶切除术与亚肺叶切除术的临床结果的数据分析和解释, 其中最大的挑战之一是减轻选择偏倚的影响。亚肺叶切除通常基于患者无法忍受肺叶切除手术来进行, 无论是由于有限的肺储备或显著的合并症。由于与非癌症相关的死亡原因, 这些患者的总体生存结果明显较差。在接受亚肺叶切除术或肺叶切除术的两组患者间的无病生存期和局部复发结果的随机化数据中期结果之前, 肿瘤等效性问题可能仍然存在争议^[15]^。

## 2 肺切缘阳性与肿瘤复发的关系

NSCLC的根治性切除术要求R0切除即显微镜下无残留。不幸的是, 5%-15%的手术切缘被发现显微镜(R1)或肉眼可见有肿瘤残留(R2)^[16, 17]^。不完全切除一直被证明是预后较差的重要原因之一, 与R0切除相比其5年生存率几乎减半^[16-20]^。由Hancock等^[19]^发表的一项研究可能是阳性切缘影响预后的最全面的例证, 该研究使用美国国家癌症数据库, 总结了2003年-2006年期间接受过根治性手术的54, 512例NSCLC患者, 切除时的分期为Ⅰ期-Ⅲ期, 结果发现5.7%的手术切缘阳性。在这些阳性切缘中, 3.1%为R1, 0.3%为R2, 其余2.3%无法根据数据库的数据确定, 发现切缘阳性患者5年总体生存率显著降低(R0 62%, R1 37%, R2 22%)。早期疾病阶段阳性切缘的影响比晚期阶段的影响更显著。

同时其他一些研究者也报道了切缘阳性与肿瘤局部复发的关系, Sawabata等^[21]^回顾了1999年-2002年间的3年病例, 研究对象包括202例接受根治性切除(肺叶和亚肺叶切除术)的早期肺癌患者, 发现R0患者的5年无病生存率为82.6%, 而边缘为阳性的患者的5年无病生存率仅为30.8%(*P*=0.001)。尽管本研究的作者承认本研究有术后化疗、放疗的干扰, 但他们仍然认为完全切除(R0)为NSCLC患者提供了更好的生存机会。Sawabata等^[22]^称手术残端肿瘤复发, 大部分发生在切缘细胞学检查结果阳性的受试者中, 此结论后来得到另一项研究的支持^[23]^。日本的佐川中号研究小组进行了一项为期5年的前瞻性随访研究, 结果表明在GGO的治疗中, 保证切缘阴性的亚肺叶切除没有任何复发, 并且术后肺功能得到很好的保留^[24]^。

近期Ajmani等^[25]^利用美国国家癌症数据库分析了早期肺癌患者行手术治疗时, 楔形切除品质与其总生存期的关系, 研究发现楔形切除术中淋巴结切除个数与手术切缘是重要的切除品质评价指标, 当切缘阴性并且切除大于5个淋巴结时, 似乎比低品质的楔形切除和立体定向放疗具有更佳的生存优势。为了确定与局部复发相关的危险因素, 杜克大学的Kelsey等^[26]^回顾了1995年-2005年期间接受肺切除术的975例Ⅰ期、Ⅱ期NSCLC患者, 多变量分析显示增加局部复发的危险因素包括鳞状/大细胞组织类型(HR=1.98)、分期 > Ⅰa(HR=2.02)、亚肺叶切除(HR=1.99)和淋巴血管浸润(HR=1.74)。当超过一种这些危险因素时会大大增加复发风险:0个、1个或2个风险因素的5年局部复发发生率分别为13%、32%和47%, 同时发现切缘阳性较阴性有更高的复发率。以上的一些报道均证实, 相较于切缘阴性, 切缘阳性的亚肺叶切除术后, 肿瘤更易局部复发。

## 3 切缘距离与切缘质量的关系

保证肺切缘阴性通常的方法是保证足够的切缘距离, 但是对于切缘距离的研究中则出现了相互矛盾的结果。其中一部分认为切缘距离有着重要的作用, Mohiuddin等^[27]^的回顾性分析发现早期NSCLC患者(T1a期病灶; 在2 cm以内)接受楔形切除治疗后的1年和2年局部复发率分别为5.7%和16.4%。在与局部复发有关的因素中, 切除范围的增加与疾病复发率呈负相关, 这一趋势在15 mm的切缘距离内保持不变, 之后效益递减, 因此认为, 对于小病灶(≤2 cm)1.5 cm是足够的切缘距离, 额外的切除并没有提供更多的收益。Sawabata等^[28]^在一项多中心前瞻性研究中提出, 当边缘距离大于最大肿瘤直径时, 边缘复发的几率较低。他们在另一项研究中指出, 外周肿瘤的肺楔形切除应保证边缘阴性, 这可通过足够的边缘肿瘤比率≥1获得, 随着边缘距离的增加, 局部复发的风险下降^[21]^。也有学者认为当NSCLC肿瘤小于或等于2 cm时, 楔形切除边缘距离的增加对于复发风险较低和总体生存期较长是一个独立的预测因素, 当切缘距离介于9 mm-11 mm之间时, 楔形切除术可能产生与肺叶切除术相当的结果^[29]^。另一项包含81例接受亚肺叶切除的Ⅰ期NSCLC患者的回顾性研究发现, 局部复发率为11.1%。与边缘≥1 cm相比边缘 < 1 cm组局部复发率增加近两倍差异(14.6 *vs* 7.5%;*P*=0.04)。值得注意的是, 远处复发无差异(< 1 cm组为14.6%, ≥1 cm组为12.5%)^[30]^。而有研究^[31, 32]^认为在对无淋巴结转移、≤3 cm的CT显示主要成分为毛玻璃影的结节行亚肺叶切除术时, 切缘距离不影响肿瘤的复发。相比之下, 切缘距离是无淋巴结转移实体型肺癌患者接受亚肺叶切除术后复发的重要危险因素。当患者心、肺功能较差时, 不适用肺叶切除术, 楔形R0切除则是Ⅰ期NSCLC手术治疗的可行选择, 切缘距离不影响复发或存活率。

切缘距离不是肿瘤局部复发的唯一危险因素, 应考虑到肿瘤大小、切缘细胞学结果、切缘距离与肿瘤大小的比值(margin/tumor size ratio, M/T)等因素^[28, 33]^。最近开展的一项针对临床Ⅰ期NSCLC高危患者的亚肺叶切除的前瞻性多中心研究(KLSG 0801)显示, 在接受亚肺叶切除的32例患者中, 3年生存率为79%, 5年无复发生存率为76%^[34]^。在KLSG 0801的子集分析中, 切缘细胞学结果和M/T比值是预后指标, 而不是切缘距离。此外, 边缘距离是早期(≤2 cm)NSCLC楔形切除患者的预后指标^[27]^。一项关于手术切缘细胞学结果的多中心前瞻性研究显示外周肺癌肺楔形切除的最佳距离是大于肿瘤大小^[33]^。Sawabata等^[28]^同样认为楔形切除外周NSCLC时必须保证切缘阴性, 其研究发现局部复发的患者均有阳性切缘, 而保证切缘阴性则必须保证M/T≥1。王益阳等^[35]^利用上海胸科医院肺癌数据库分析了2010年-2015年共746例行楔形切除的患者, 发现当楔形切除肺组织体积越大和/或肿瘤体积越小, 总生存和肺癌症状评分越好。如果体积比达到10:1或更高, 则3年存活率约为90%。以上的相关报道介绍了不同的切缘距离与切缘质量的关系, 保留更多健康肺组织与保证切缘阴性似乎是难以调和的矛盾, 关于切缘距离的争论目前仍在持续, 而把控切缘距离的, 是由手术医生在术中决定, 因此需要更多的手术医生报道关于切缘距离的研究。

## 4 检测肺切缘的方法

NSCLC的手术切除原则之一是彻底切除肿瘤, 这其中就包含保证切缘阴性。确定是否为R0切除术最常用的方法是术中冰冻切片检查, 然而由于存在假阴性和假阳性, 冰冻切片也被认为是不完善的。虽然假阴性和假阳性率通常低于5%^[36]^, 但也有报道称假阴性率可高达41.7%^[37]^, 原因包括抽样误差(切除的标本切缘上偶尔有孤立的癌细胞^[38]^)和读片错误(将恶性细胞误认为淋巴细胞或腺体, 将原位病变误认为化生组织以及腐蚀物模糊浸润癌等)等。而残留于切缘的癌细胞, 大约50%的可能导致术后切缘肿瘤复发^[39, 40]^。此外, 一些研究^[41]^表明, 使用冷冻切片, 特别是在支气管边缘, 很少有阳性切缘的检查结果。根据这些信息, NCCN指南指出, 在进行解剖性肺切除术时不建议进行冰冻切片分析切缘^[42]^。

研究表明术中冰冻可能无法准确无误地验证切缘, 可以通过额外的细胞学和分子学方法评估切缘以获得更多信息, 这种方法是由Sawabata等^[43, 44]^提出的。他们发现, 即使在R0切除后, 局部复发也可能发生。为了更好地理解这种生物学行为, 他们又评估了30例接受楔形切除的周围型肺癌患者的手术标本, 除了获得组织学评估切缘作为充分切除的指标之外, 他们还使用细胞学方法来评估切缘^[22]^, 后者是通过刮擦和灌洗液获得切缘细胞学样品^[44]^。结果在40%的组织学阴性的切缘中, 发现了恶性肿瘤的细胞学证据^[44]^。Masasyesva等^[45]^报道了一种使用分子测试法检测切缘是否受癌累及的新方法, 他们回顾性分析了44例接受R0亚肺叶切除术治疗NSCLC患者的病理检查标本, 从中发现13例存在12例表达*k-ras*的突变密码子, 然后通过荧光间隙连接酶链反应评估这13例患者标本的切缘, 通过分子评估, 在这13例中9例患者具有*k-ras*突变的细胞。临床随访结果发现9例中的6例患者最终发展为局部复发, 切缘没有癌累及分子证据的患者则没有出现疾病复发。而在这个实验中, 疾病复发是否有其他突变基因的干扰, 我们无从得知, 期待更多、更深入的研究。尽管如此, Sawabata和Masasyesva的工作仍旧提供了具有启发性的结论, 即传统组织学检测方法(标准冰冻切片和常规病理检测)可能只是对切缘的粗略评估。

## 5 现状与展望

由于早期NSCLC患者的逐年增多, 使得胸外科医生在手术方式和范围的选择中有了更多的余地。为了保证完整切除, 在治疗过程中对外科医师提出了更高的要求, 不仅要保证R0切除, 更重要的是要保证切缘“真正意义”上的无肿瘤细胞残留。一方面要提高外科医师对切缘阴性重要性的认识, 另一方面也要求相关辅助科室使用多种检查方法, 加强对切缘的检验, 避免假阴性的出现。根据浙江大学医学院附属第一医院胸外科同仁的大样本分析, 早期NSCLC在保证组织学检查切缘阴性的前提下, 可以使用亚肺叶切除, 而传统的术中冰冻检查, 由于取材部位、冰冻制片技术、读片医师资历参差不齐等因素的影响, 并没有想象中的可靠。未来可能会有更多的关于肺切缘的回顾性分析和随机对照实验, 从而制定出针对手术方式、切缘距离和检测标准等更加合理并有效的准则。

## References

[b1] 1China's latest cancer data for 2017. Zhongguo Zhong Liu Lin Chuang Yu Kang Fu, 2017, 24(5): 574.2017年中国最新癌症数据. 中国肿瘤临床与康复, 2017, 24(5): 574.

[2] Baisi A, Raveglia F, De S M (2015). Do margins really affect prognosis in wedge resection for early-stage lung cancer?. Ann Thorac Surg.

[3] Goldstraw P, Crowley J, Chansky K (2007). The IASLC Lung Cancer Staging Project:proposals for the revision of the TNM stage groupings in the forthcoming (seventh) edition of the TNM classification of malignant tumours. J Thorac Oncol.

[4] Ginsberg RJ, Rubinstein LV (1995). Randomized trial of lobectomy versus limited resection for T1 N0 non-small cell lung cancer. Ann Thorac Surg.

[5] Hoffmann TH, Ransdell HT (1980). Comparison of lobectomy and wedge resection for carcinoma of the lung. J Thorac Cardiovasc Surg.

[6] Jacobs CD, Jafari ME (2017). Early results of lung cancer screening and radiation dose assessment by low-dose CT at a community hospital. Clin Lung Cancer.

[7] Simmons VN, Gray JE, Schabath MB (2017). High-risk community and primary care providers knowledge about and barriers to low-dose computed topography lung cancer screening. Lung Cancer.

[8] Aberle DR, Adams AM, Berg CD (2011). Reduced lung-cancer mortality with low-dose computed tomographic screening. N Engl J Med.

[9] Cao J, Yuan P, Wang Y (2018). Survival rates after lobectomy, segmentectomy and wedge resection for the non-small cell lung cancer. Ann Thorac Surg.

[10] Landreneau RJ, Sugarbaker DJ, Mack MJ (1997). Wedge resection versus lobectomy for stage Ⅰ (T1 N0 M0) non-small-cell lung cancer. J Thorac Cardiovasc Surg.

[11] Donington JS (2015). Survival after sublobar resection versus lobectomy for clinical stage ⅠA lung cancer:analysis from the national cancer database. J Thorac Oncol.

[12] Okada M, Koike T, Higashiyama M (2006). Radical sublobar resection for small-sized non-small cell lung cancer:a multicenter study. J Thorac Cardiovasc Surg.

[13] Xu S, Liu YJ (2017). New Progress in the study of glass grinding in the lungs. Lin Chuang Fei Ke Za Zhi.

[14] Cao C, Chandrakumar D, Gupta S (2015). Could less be more?-A systematic review and *meta*-analysis of sublobar resections versus lobectomy for non-small cell lung cancer according to patient selection. Lung Cancer.

[15] Cao C, Tian DH, Wang DR (2017). Sublobar resections-current evidence and future challenges. J Thorac Dis.

[16] Riquet M, Achour K, Foucault C (2010). Microscopic residual disease after resection for lung cancer:a multifaceted but poor factor of prognosis. Ann Thorac Surg.

[17] Wind J, Smit E J, Senan S (2007). Residual disease at the bronchial stump after curative resection for lung cancer. Eur J Cardiothorac Surg.

[18] Ghiribelli C, Voltolini L, Paladini P (1999). Treatment and survival after lung resection for non-small cell lung cancer in patients with microscopic residual disease at the bronchial stump. Eur J Cardiothorac Surg.

[19] Hancock JG, Rosen JE, Antonicelli A (2015). Impact of adjuvant treatment for microscopic residual disease after non-small cell lung cancer surgery. Ann Thorac Surg.

[20] Schuchert MJ, Abbas G, Awais O (2012). Anatomic segmentectomy for the solitary pulmonary nodule and early-stage lung cancer. Ann Thorac Surg.

[21] Sawabata N, Maeda H, Matsumura A (2012). Clinical implications of the margin cytology findings and margin/tumor size ratio in patients who underwent pulmonary excision for peripheral non-small cell lung cancer. Surg Today.

[22] Sawabata N, Matsumura A, Ohota M (2002). Cytologically malignant margins of wedge resected stage Ⅰ non-small cell lung cancer. Ann Thorac Surg.

[23] Higashiyama M, Kodama K, Takami K (2003). Intraoperative lavage cytologic analysis of surgical margins in patients undergoing limited surgery for lung cancer. J Thorac Cardiovasc Surg.

[24] Sagawa M, Oizumi H, Suzuki H (2018). A prospective 5-year follow-up study after limited resection for lung cancer with ground-glass opacity. Eur J Cardiothorac Surg.

[25] Ajmani GS, Wang CH, Kim KW (2018). Surgical quality of wedge resection impacts overall survival in patients with early stage non-small cell lung cancer. J Thorac Cardiov Sur.

[26] Kelsey CR, Marks LB, Donna Hollis MS (2009). Local recurrence after surgery for early stage lung cancer. Cancer.

[27] Mohiuddin K, Haneuse S, Sofer T (2014). Relationship between margin distance and local recurrence among patients undergoing wedge resection for small (≤2 cm) non-small cell lung cancer. J Thorac Cardiovasc Surg.

[28] Sawabata N, Ohta M, Matsumura A (2004). Optimal distance of malignant negative margin in excision of non-small cell lung cancer:a multicenter prospective study. Ann Thorac Surg.

[29] Wolf AS, Swanson SJ, Yip R (2017). The impact of margins on outcomes after wedge resection for stage Ⅰ non-small cell lung cancer. Ann Thorac Surg.

[30] El-Sherif A, Fernando HC, Santos R (2007). Margin and local recurrence after sublobar resection of non-small cell lung cancer. Ann Surg Oncol.

[31] Moon Y, Lee KY, Moon SW (2017). Sublobar resection margin width does not affect recurrence of clinical N0 non-small cell lung cancer presenting as GGO-predominant nodule of 3 cm or less. World J Surg.

[32] Maurizi G, D'Andrilli A, Ciccone AM (2015). Margin distance does not influence recurrence and survival after wedge resection for lung cancer. Ann Thorac Surg.

[33] Sawabata N, Maeda H, Matsumura A (2012). Clinical implications of the margin cytology findings and margin/tumor size ratio in patients who underwent pulmonary excision for peripheral non-small cell lung cancer. Surg Today.

[34] Onishi H, Araki T (2013). Stereotactic body radiation therapy for stage Ⅰ non-small-cell lung cancer:a historical overview of clinical studies. Jpn J Clin Oncol.

[35] Wang Y, Rui W, Zheng D (2017). Predicting the recurrence risk factors and clinical outcomes of peripheral pulmonary adenocarcinoma ≤3 cm with wedge resection. J Cancer Res Clin Oncol.

[36] Maygarden SJ, Detterbeck FC, Funkhouser WK (2004). Bronchial margins in lung cancer resection specimens:utility of frozen section and gross evaluation. Mod Pathol.

[37] Kaiser LR, Fleshner P, Keller S (1989). Significance of extramucosal residual tumor at the bronchial resection margin. Ann Thorac Surg.

[38] Sawabata N, Karube Y, Umezu H (2008). Cytologically malignant margin without continuous pulmonary tumor lesion:cases of wedge resection, segmentectomy and lobectomy. Interact Cardiovasc Thorac Surg.

[39] Sawabata N, Matsumura A, Ohota M (2002). Cytologically malignant margins of wedge resected stage Ⅰ non-small cell lung cancer. Ann Thorac Surg.

[40] Higashiyama M, Kodama K, Takami K (2003). Intraoperative lavage cytologic analysis of surgical margins in patients undergoing limited surgery for lung cancer. J Thorac Cardiovasc Surg.

[41] Owen RM, Force SD, Gal AA (2013). Routine intraoperative frozen section analysis of bronchial margins is of limited utility in lung cancer resection. Ann Thorac Surg.

[42] National Comprehensive Cancer Network Clinical Practice Guidelines. Surgical Therapy in non-small cell lung cancer.(version 7.2015).

[43] Sawabata N, Mori T, Iuchi K (1999). Cytologic examination of surgical margin of excised malignant pulmonary tumor:methods and early results. J Thorac Cardiovasc Surg.

[44] Utsumi T, Sawabata N, Inoue M (2010). Optimal sampling methods for margin cytology examination following lung excision. Interact Cardiovasc Thorac Surg.

[45] Masasyesva BG, Tong BC, Brock MV (2005). Molecular margin analysis predicts local recurrence after sublobar resection of lung cancer. Int J Cancer.

